# Elbow Dislocation with Irretrievable Rotating‐Rope Injury of the Forearm: Case Report and Literature Review

**DOI:** 10.1111/os.13234

**Published:** 2022-02-22

**Authors:** Jian‐chuan Wang, Song Qin, Zong‐pu Wang

**Affiliations:** ^1^ Department of Orthopaedics Affiliated Zhongshan Hospital of Dalian University Dalian China

**Keywords:** Cross damage, Essex‐Lopresti injury, Interosseous membrane, Urotary noose upper and lower radioulnar joint

## Abstract

**Background:**

Simultaneous dislocation of the elbow, radioulnar joint and proximal radius fracture with rotary noose injury to the medial ulna tubercle is extremely rare. An emergency surgery was performed to reduce it. The radial head with the backbone was reset after two hammers were fixed, then the radial capitulum safety was fixed with a locking plate. After the ulnar instability was examined, two Kirschner wires were drilled percutaneously to fix the elbow flexion at 100° under closed reduction, and two Kirschner wires were drilled percutaneously to fix the ulnar joint. Good follow‐up results were achieved. To the best of our knowledge, this is the first report on this particular type of injury and on this approach to treating this type of injury.

**Case presentation:**

We report the case of a 36‐year‐old male, who extended and landed on his left hand to protect his child in right arm before felling, resulting in severe pain and deformity of his left elbow and wrist and loss of movement in these joints. X‐ray examination found proximal distal radioulnar joints, a proximal radial fracture and a dislocation bowstring in the ulna nodule. For a timely diagnosis in an emergency open reduction situation, accurate judgment of this injury is highly important. After 12 months of postoperative follow‐up, the patient was symptom‐free, and radiographs showed fracture healing.

**Conclusion:**

We performed emergency reduction and internal fixation of the elbow and successfully saved elbow function, no stability decrease and movement restriction. This case also provides a new reference for the treatment of this type of elbow fracture dislocation.

## Graphical abstract

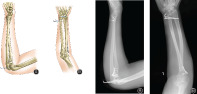

(A, B)Anatomical reduction of radial head, fixation with two headless nails and locking plates, physical examination of instability of radioulnar joint, supination of percutaneous fixation with two Kirschner wires, elbow instability, elbow flexion 90 degrees fixation of ulnar joint.

## Introduction

The elbow joint is composed of the lower end of the humerus and the upper end of the ulna and radius. It is a compound joint that is composed of the humerus joint, the humerus radial joint and the proximal radioulnar joint within the same joint capsule. The elbow joint is the second most common site of dislocation injury after the glenohumeral joint. Elbow dislocation is a common injury in clinical practice, which is treated by orthopedic surgeons. Patients with a dislocated joint are usually managed in the emergency room because of the severe pain and functional impairment caused by such an injury. Early treatment consists of simple elbow dislocation reduction and fixation for less serious cases. However, if radial head fractures, elbow dislocation or ulna olecranon fractures are observed, these complex joint injuries become even more complicated, as there is a risk of traumatic arthritis. Heterotopic ossification can have serious consequences, such as loss of function and joint stiffness, which will be a source of continual pain for patients. Elbow dislocation with forearm injury is often missed in the emergency room, so elbow dislocation with radial head fracture should be highly suspected in cases of forearm and radioulnar injuries. Traumatic distal radioulnar joint instability is commonly associated with Colle's type fractures, Smith's type fractures^1^, ^2^, radial shaft fractures (Galeazzi's fracture dislocation), radial head fractures, and elbow dislocation^3^, ^4^. Geiger's rare and Montesian fractures are classically described in forearm injuries, accounting for 3%–6% and 1%–2% of forearm fractures, respectively^5^. A rare injury associated with distal radioulnar joint dislocation of the radial head or neck fracture and rupture of the interosseous membrane is known as Essex‐Lopresti injury^6^. Another rare injury was reported for the first time in 2002 by Leung^7^, who described cross‐joint damage with upper and lower radial scale joint dislocation or subluxation occurring simultaneously and associated with the radial head or ulna without ipsilateral ulnar styloid process fracture of radial fractures or loss of interosseous membrane integrity. The diagnosis was confirmed by imaging the X‐ray side of the foot. We report simultaneous elbow proximal radial ulnar distal radial joint dislocation and fracture of the radial head and neck simultaneous to rotation of the noose in the ulna nodule; this damage is different from Essex‐Lopresti forearm fracture dislocation cross damage. Accurate diagnosis and treatment of the condition is challenging, and misdiagnosis can easily occur. Additionally, missed diagnosis can lead to persistent pain and wrist instability. Early diagnosis and timely treatment help the patient achieve the best results.

## Case Report

**Fig. 1:**
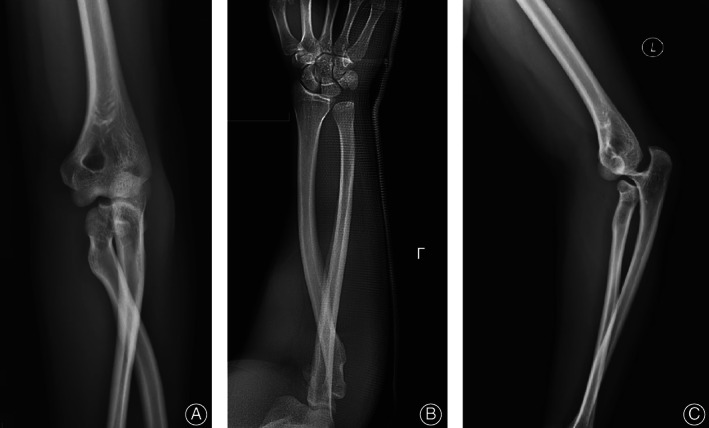
(A, B) The anteroposterior position of the elbow joint shows the position of the humeroulnar and the humeroradial relationship. The proximal end of the radius is shifted to the medial side, and the joint with the medial humericulocephalus is presented in a cross image. (C) Lateral view showing complete dislocation of the elbow with intersection of the proximal ulna and radius

**Fig. 2:**
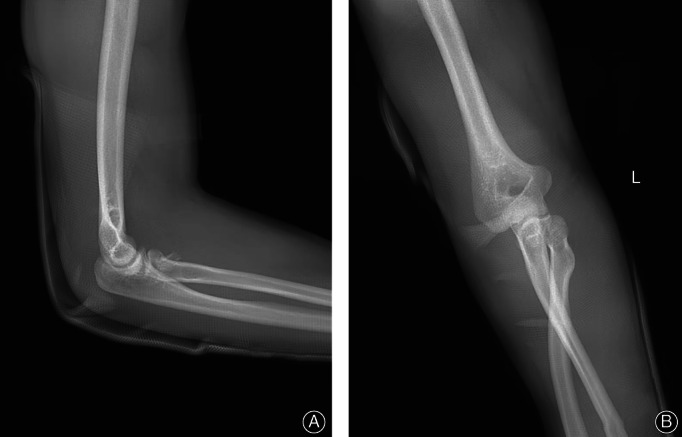
**(**A) Lateral view of the elbow after reduction showing that the ulnar joint returned to normal. (B) Anteroposterior radiographs after reduction showed recovery of the ulnar joint, asymmetry of the brachioradial joint, and displacement of the proximal radius to the medial side of the ulna

**Fig. 3:**
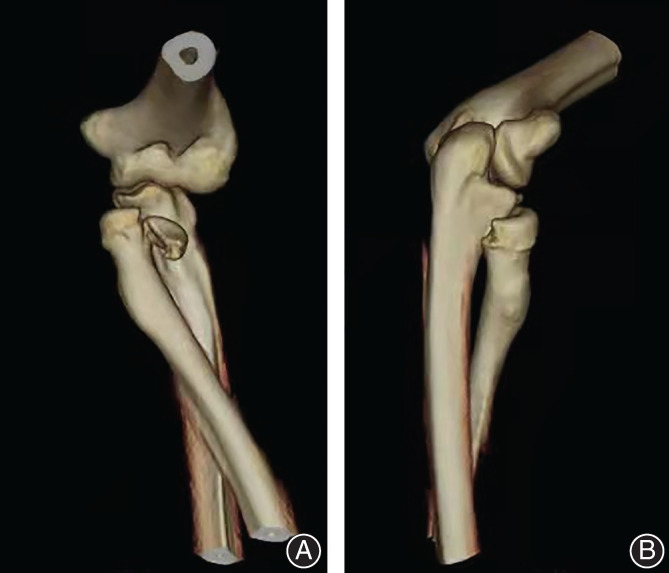
(A) Three‐dimensional CT of the elbow showing dissociation and displacement of the radial head but no dissociation or displacement of the radial neck fracture. (B) Behind the elbow, the radial head rotates the noose to the ulnar tuberosity more clearly

A 36‐year‐old male was holding his child after drinking alcohol and complained that he lost his balance while playing and spinning around. As he fell, to protect the child in his right arm, he extended and landed on his left hand, resulting in severe pain and deformity of his left elbow and wrist and loss of movement in these joints. At the time of the injury, the elbow was flexed, and the wrist was in an extended position. Movement and sensation of the fingers were normal. No neurovascular injury was observed. The patient was admitted to the emergency department at a tertiary general hospital near his home, and radiographs were taken of the forearm including the wrist and elbow (Fig. 1). Radiographs showed dislocation of the proximal and distal radioulnar joint, dislocation of the radial head, and neck fracture of the rotated noose in the ulnar tuberosity posterior to the humerus and humerus radioulnar relationship. Manual reduction was performed by the emergency physician under brachial plexus anesthesia in the orthopedic emergency department, but the initial reduction failed. The radioulnar joint of the radioulnar humerus remained dislocated. The senior director of the orthopedic department performed the second reduction under general anesthesia and reexamined the imaging X‐ray after the procedure. The results showed that the dislocation of the elbow was corrected, but the position of the radius had not been restored (Fig. 2). The patient was advised to receive further treatment after plaster fixation, but the patient refused and came to our hospital. Emergency CT examination of the elbow was performed. CT showed a fracture of the radial head and neck, in which a fragment of the head was detached, and the rotational noose of the radial head and neck was posterior to the ulnar tubercle (Fig. 3). At this time, the anesthesia had worn off, and the patient complained of elbow pain and the sensation of a foreign body in the joint. The elbow was already swollen after two repositions, and the skin tension was tolerable.

## Surgical Methods

**Fig. 4:**
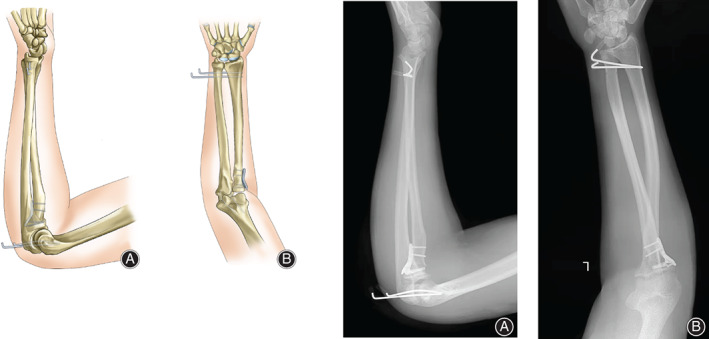
Surgical schematic diagram (A, B): Postoperative anteroposterior and lateral films. The elbow dislocation was reset, followed by the forearm rotation noose proximal ulnar radial. Two headless screws were applied to the radial fracture. To restore the humerus feet and humerus oar, gram needle percutaneous fixation was applied at 90 degrees elbow flexion. At the same time, the forearm supination ulnar radial joints underwent percutaneous fixation

After confirming the absence of surgical contraindications, we communicated with the patient and his family and consent for surgery was obtained. Following examination, the patient was admitted for emergency surgery. Radial head and neck fractures were discovered, and the radial proximal noose was located behind the ulna nodules. The periosteum was stripped, and the proximal radius was pried. The radius of the bowstring proximal was observed to bounce quickly. The surgeon visually confirmed that the interosseous membrane was intact and that the radial head was fractured. The two hammers were fixed, the radial head with the backbone was reset, and the radial capitulum safety was fixed with a locking plate. The ulnar instability was examined, two Kirschner wires were drilled percutaneously to fix the elbow flexion at 100° under closed reduction, and two Kirschner wires were drilled percutaneously to fix the ulnar joint. The intraoperative fluoroscopic position was good. Surgical schematic diagram (Fig. 4)

## Postoperative Functional Exercise and Follow‐Up

**Fig. 5:**
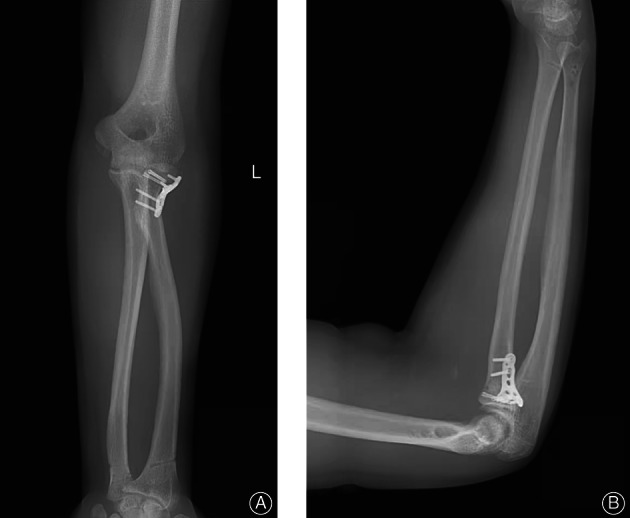
An ultra‐elbow and wrist plaster was used to flex the elbow by 90°, and the wrist was fixed in the supine position for 6 weeks. X‐rays were reexamined every week to monitor the fracture position. Orthopedic rehabilitation exercises were performed twice a week from 4 weeks to 6 months after the operation.(A, B). After 12 months of postoperative follow‐up, the patient was symptom‐free, and radiographs showed fracture healing

An ultra‐elbow and wrist plaster was used to flex the elbow by 90°, and the wrist was fixed in the supine position for 6 weeks. X‐rays were reexamined every week to monitor the fracture position. Orthopedic rehabilitation exercises were performed twice a week from 4 weeks to 6 months after the operation. After 12 months of postoperative follow‐up, the patient was symptom‐free, and radiographs showed fracture healing (Figure 5). The forearm was pronated, and supination was fixed at 75°. The elbow flexion angle ranged from 0° to 140°, and the wrist range of motion reached 80° and 70°. The patient was able to return to work without any significant functional limitations.

The visual analogue score (VAS) was measured before and after surgery. The Disabilities of the Arm and Shoulder and Hand (DASH) score was 10 points, with a higher score indicating greater pain. Upper limb function was rated on a scale of 0 to 100, with a lower score indicating better function. The VAS score at the last postoperative follow‐up was 1.718 ± 0.65, which was significantly lower than that before surgery (7.86 ± 1.45) (*P* < 0.05). The DASH score after the operation decreased from 87.12 ± 7.64 to 52.46 ± 9.26 (*P* < 0.05).

## Discussion

The elbow joint is the second most common upper‐limb joint to experience dislocation injury after the glenohumeral joint, with a reported incidence of 5.2 cases per 100,000 people^8^. If fracture occurs in the case of a dislocated elbow, it is regarded as a complex elbow dislocation. These injuries usually require a higher level of treatment, and studies have shown that open reduction and internal fixation or radial head replacement for elbow fractures and dislocations can achieve the same range of motion and reliable results^9^. However, no cases of elbow dislocation combined with radial head and neck fracture proximal radioulnar joint injury combined with proximal radial fracture with rotatory noose on the medial side of the ulnar tubercle have been reported. Considering the forearm as a unit rather than as a single structure is crucial to understanding Essex‐Lopresti injury. The forearm includes the proximal radius and ulnar joint, the distal radioulnar joint, and the interosseous membrane. Essex‐Lopresti fracture is a complex injury in which longitudinal force is transmitted through the wrist to the radial head and, if sufficient force is applied, a continuous injury can occur. It is characterized by dislocation of the distal radioulnar joint (DRUJ) with fracture of the radial head and rupture of the interosseous membrane^10^. Typically, in Essex‐Lopresti injuries, attention is focused on radial head injuries, with distal radioulnar displacement being ignored during the initial examination. If distal radioulnar instability is not adequately recognized and managed, chronic wrist symptoms may develop, such as pain and instability^11^. A number of different types of Essex‐Lopresti injuries have been reported in the literature, accompanied by unilateral or bilateral elbow dislocation of the radial head and neck fracture and distal radial displacement of the interosseous membrane rupture^12^. However, there have been no reported cases of elbow dislocation with distal and proximal radioulnar joint dislocation in which the rotatory noose of the proximal radius is behind the ulnar tubercle and the interosseous membrane is intact. Cross forearm injuries with simultaneous dislocation or subluxation of the radioulnar joint are associated with radial head or ulnar styloid fractures and an intact interosseous membrane but not with elbow dislocation and rotational noose of the proximal radius. Cross forearm injuries produce a cross‐shaped appearance on lateral radiographs, sometimes even on anteroposterior radiographs.

Essex‐Lopresti injuries mainly manifest as forearm instability caused by longitudinal cleavage of the interosseous membrane of the forearm. Most of the literature has focused on fractures of the radial head, and damage to the inferior radioulnar joint and the interosseous membrane has been neglected^13^. There has also been no diagnostic manifestation of Essex‐Lopresti lesion in cross injuries in which the interosseous ligament structure is remarkably intact, including separation of the forearm bone before and after the displacement of the proximal radius and the acute phase of excessive tenderness and swelling of the forearm. In previous studies, only seven adult cases with simultaneous dislocation of the radial head and distal radioulnar joint without fracture were recorded^14^. Cross injuries highlight the importance of injuries at the other end of the radioulnar joint, which are also easily overlooked, followed by delayed or neglected distal or proximal radioulnar joint dislocations, for which various stabilizing procedures can be performed^15^. In a study of 12 patients with acute ELI, Grassmann *et al*. concluded that early reduction and temporary fixation of the radioulnar joint with Kirschner wires resulted in satisfactory interim results^16^.

The case we report herein is completely different from the Essex‐Lopresti injury and the cross injury, in which the entire rotation, flexion and extension mechanisms of the forearm are damaged. In our case, there was dislocation of the elbow, an interchange of the ulnar and radial positions, and more importantly, fracture of the proximal radius, similar to the Bosworth fracture. No amount of rotational flexion and extension could reduce the proximal radius of the rotated noose. Preoperative noose‐like pain was considered to be caused by the noose proximal radius on the medial side of the ulnar tubercle. If the tension of the interosseous membrane was lost, the noose proximal radius would not be behind the ulna, and it could be reduced by rotation. Intraoperatively, we also confirmed the integrity of the forearm interosseous membrane. Therefore, the purpose of this clinical case is to improve emergency department nurses' awareness of this rare injury by explaining the rare diagnosis and treatment of such patients, confirmed with the necessary imaging examination to avoid blind restoration, closed reduction and failure from attempting to correct the damage by trial and error. Fractures like the Bosworth ankle‐joint injury usually cannot be treated by closed reduction, and too many attempts can further aggravate the injury and cause severe complications.

We share this case in the interest of educating orthopedic surgeons about this rare and complex compound dislocation and fracture. In this case, the first physician to see the patient in the emergency room could have immediately ordered a CT scan and 3D reconstruction or MRI in addition to X‐ray to further assess injuries to the ligaments and soft tissues. While the most basic imaging examination is essential to determine the dislocation direction of the fracture, CT can further determine the presence of fractures hidden by the dislocation in X‐rays. Upon confirmation, the first reduction should be carried out in an operating room under general anesthesia to fully relax the muscles, rather than in the emergency room where the focus will be to quickly address the injury, which will increase pain and fear in patients and make subsequent treatment more difficult. If the reduction is not successful, health care providers should immediately adopt an open reduction plan. Any dislocated joint needs to be reduced as a first treatment. The joint should not be fixed in the dislocated state, which could greatly reduce joint function. During open reduction, examination of adjacent joints, especially limbs on the same axis, should be performed. The radioulnar joint should be examined for radial head fracture, and the ulnar brachioradial joint should be examined for reduction of elbow dislocation. When elbow dislocation is combined with radial head fracture, the ulnar brachioradial relationship should be examined not only for the upper ulnar brachioradial relationship but also for the subcapsular ulnar joint of the forearm. Joint dislocation damages the surrounding tendinous structures. After osseous structure damage reduction, tendinous structures also need time to heal, as the fixed bony structure requires the protection of tendinous structures. Thus, after joint stability is restored with rehabilitation exercises, the patient will have stable and flexible joints rather than unstable joints.

Due to our inexperience in the treatment of this rare injury, we regrettably did not complete elbow MRI to evaluate the elbow ligament. The bony structure consists of the radial head proximal to the ulna and the distal humerus. The soft tissue structure is mainly the elbow capsule, elbow ligaments and muscle tendinous tissue, and elbow valgus stability is primarily maintained by the radial head and medial collateral ligament. The medial collateral ligament fights valgus stress and provides support for the ulnar joint of the elbow. When the medial collateral ligament is not functional, valgus relaxation and pain can occur on the inside of the elbow. The primary function of the lateral collateral ligament is to stabilize the annular ligament proximal to the humerus and ulna to maintain a normal relationship between the proximal forearm and the trochlea of the humerus and the capitulum. After concentric reduction of acute elbow dislocation, the lateral collateral ligaments often heal and recover well. The lateral collateral ligaments can be healed with elbow movement and often with appropriate tension. Therefore, when encountering such a rare case in clinical work, emergency physicians should immediately conduct full imaging examinations, comprehensively evaluate bone and ligament injury, and formulate a complete diagnosis and treatment plan to maximize the recovery of joint function and reduce the patient's pain.

## Conclusion

We report a rare injury involving simultaneous dislocation of the elbow, proximal radiulnar joint, distal radiulnar joint and proximal radius fracture with a rotating noose on the medial side of the ulnar tuberosity. For a rapid and timely diagnosis in an emergency open reduction situation, early diagnosis and treatment can result in the best curative effect. This case also provides a new reference for the treatment of this type of elbow fracture dislocation.
